# mHealth Uses and Opportunities for Teens from Communities with High Health Disparities: A Mixed-Methods Study

**DOI:** 10.1007/s41347-022-00278-y

**Published:** 2022-09-13

**Authors:** Colleen Stiles-Shields, Karen M. Reyes, Joseph Archer, Nia Lennan, Jim Zhang, Wrenetha A. Julion, Niranjan S. Karnik

**Affiliations:** 1 Rush University Medical Center, Chicago, IL USA; 2 Present Address: University of Illinois at Chicago, Chicago, IL USA; 3 Rosalind Franklin University of Medicine and Science, North Chicago, IL USA

**Keywords:** Teen, Health disparities, mHealth, Caregiver, Community

## Abstract

Despite widespread access to smartphones, teens from communities facing significant behavioral health disparities typically have low mobile health (mHealth) engagement. The purpose of this study was to characterize teen and caregiver perspectives about smartphone use and access, mHealth, and how mHealth could address teens’ behavioral health needs during the pandemic and beyond. Remote recruitment and methodologies were used to engage 17 teens (M age = 15.9 ± 0.9) and 10 caregivers living in urban communities with significant socioeconomic and health disparities. Participants completed a focus group or interview session (based on preference) and self-report questionnaires (e.g., behavioral health history, pandemic impacts, technology use). Qualitative and quantitative data were analyzed using thematic and descriptive analyses, respectively. Both quantitative and qualitative data indicated relevant behavioral health concerns for teens and their families, impacts from the pandemic, and frequent smartphone use. Primary teen and caregiver themes included (1) health and wellness concerns, (2) barriers, (3) use of smartphones, (4) impacts of smartphones, and (5) opinions/suggestions for mHealth. This multi-method and multi-informant study highlighted the lived experiences of teens from marginalized communities and offered key insights to increase the acceptability and real-world engagement of mHealth tools. To address barriers to care for this population beyond the pandemic, clear messaging must be used for mHealth tools (e.g., data privacy, expectations of use). These findings testify to the importance of collaboration with teens and caregivers from communities facing large health disparities in future mHealth design, development, and deployment.

Mobile health (mHealth) is likely to remain as a permanent form of care delivery and must therefore be conducted inclusively and intentionally to avoid exacerbating disparities in healthcare access (Figueroa & Aguilera, 2020; Schueller et al., 2019). Human-centered design (HCD) utilizes mixed-methods approaches to design technology alongside potential users, matching the context of use to their everyday lives (d. School, 2010; IDEO, 2015; International Organization for Standardization, 2019). While this methodology sounds inclusive in theory, the practice has been historically framed within structural racism (Hill et al., 2015; Iskander, 2018; Safavi et al., 2019). To become a more equitable delivery mechanism, mHealth developed with HCD must be re-examined from a lens of equity, inclusion, and anti-racism (Kendi, 2019). A first step in this process is collaborating with minoritized populations to better understand how mHealth may fit into their lives and serve their needs (Baum et al., 2006; Stiles-Shields et al., 2022).

Most youth with anxiety or depression do not receive treatment (SAMHSA, 2014). The greatest systemic barriers to care are experienced by teens with socially complex needs (SCN). That is, teens who may face overlapping adversities (e.g., adverse childhood events [ACEs], pediatric conditions) and/or marginalized identities (e.g., racial, ethnic, gender, and/or sexual minoritized identity populations; Bounds et al., 2020). Given the ubiquity of smartphone access for youth (Anderson & Jiang, 2018), mHealth interventions—and broader digital mental health interventions and tools—have been purported as a likely way to access care (Anderson-Lewis et al., 2018; Kumar et al., 2019). However, smartphone ubiquity has not equated to mHealth use and engagement in real-world settings, particularly with young populations with socially complex needs.

Barriers to mHealth use and engagement in real life are multi-factored. First, despite excellent examples of co-design and other collaborative processes with youth for digital design (Fleming et al., 2016; Hetrick et al., 2018; Thabrew et al., 2018), the majority of mHealth tools have been designed for and with adults (Noorbergen et al., 2021; Psihogios et al., 2022) and populations with more privilege and/or access to opportunities for care (Safavi et al., 2019; Stiles-Shields et al., 2020). Namely, teens with SCN have rarely been included in design, evaluation, or deployment strategies for mHealth. Second, despite universal endorsement of access to smartphones (Anderson & Jiang, 2018), consistency of access may be variable. For example, teens with SCN may share a phone with a member of their household have phone privileges revoked as punishment or experience service outages. Third, while apps are abundant in the marketplace, it is difficult for teens to determine which are credible, evidence-based, and/or appropriate for their needs (Neary & Schueller, 2018; Psihogios et al., 2020). Finally, despite the heightened awareness of the importance of care that is culturally competent and inclusive prior to and during the pandemic (Benuto et al., 2019; Fortney et al., 2015; Hilty et al., 2021a, b; Winters et al., 2019), implementation of mHealth into formal behavioral health care systems for teens has lagged (Psihogios et al., 2022). In sum, teens with SCN have been systematically overlooked or excluded from mHealth design, evaluation, and implementation, likely perpetuating low real-world engagement with mHealth for these youth.

The purpose of the current study was to harness HCD methodologies to identify the needs of teens with SCN who may access mHealth to improve mental and behavioral health. Participants were recruited from the West and South Side Communities of Chicago, Illinois, USA, where many residents experience stark health disparities, violence exposure, and higher economic hardship compared with other communities in the city (Lange-Maia et al., 2018; Pierce et al., 2021). Data were collected during the COVID-19 pandemic, with the intent to inform general practice moving forward.

## Methods

### Participants

The study was approved by the Institutional Review Board and all participants provided informed consent. Following eligibility screening, potential participants were sent secure consent/assent materials via REDCap and completed informed consent (caregiver participants and/or consent for their teen’s participation) and/or assent (teen participants) with a research staff member on the phone. Caregivers of teen participants were able to provide parental consent in Spanish or English.

Eligible participants were (1) a teen (12–17 years old) or a guardian of a child (12–17 years old), (2) residents of a West or South Side Community, where many residents experience stark health disparities, violence exposure, and higher economic hardship compared with other communities in the city (Lange-Maia et al., 2018; Pierce et al., 2021), (3) smartphone users within the past week, (4) able to speak and read in English, and (5) without self-reported presence of a psychiatric disorder that would make participation inappropriate/dangerous (e.g., active psychosis). Participants were compensated for their time with a $15 gift card.

### Procedure

Participants could opt to participate in an individual interview or a focus group. Sessions were conducted using a HIPAA-compliant Zoom account with secure, password-protected links. Focus group sessions (1) were conducted with teens and caregivers separately, (2) had no more than five participants at a time, and (3) lasted 45–60 min. The sessions were led by researchers who identify as BIPOC or mixed race and were supervised by a licensed psychologist. All groups were asked a standard set of questions, adapted from a previous study assessing technology use in homeless youth (Adkins et al., 2017). All focus group/interview sessions were audio recorded for transcription and data analyses.

### Measures

Self-report assessments were administered and managed via REDCap (Harris et al., 2009). Participants self-reported demographic characteristics (e.g., age, gender, race); caregivers also reported about their teen. Participants were also queried about previous personal (teen) or family (caregiver) psychiatric and/or medical diagnoses and their subsequent treatment history.

The Media and Technology Usage and Attitudes Scale (MTUAS) was administered to all participants to assess media and technology use (Rosen et al., 2013). The MTUAS usage and attitudes subscales demonstrated acceptable reliability for the teen (αs = 0.60–0.90) and caregiver reports (αs = 0.67–0.98).

### Data Analysis

Qualitative data from the focus group and interview sessions were analyzed using thematic analysis (Braun & Clarke, 2006), with an inductive coding methodology (Patton, 1990). Coding was concurrent with focus group sessions so as to determine the number of groups required to reach data saturation. The first through fifth authors worked as coders, with each session initially coded by two independent coders. Candidate themes were then created via group consensus. Upon the completion of data collection, group consensus was used to create final themes and subthemes. Quantitative data from the self-report questionnaires were examined via descriptive analyses.

## Results

### Participants

Eight focus groups and seven individual interviews were completed. The sample consisted of 17 teens (82.4% cisgender female) and 10 caregivers (all biological parents; 90% cisgender female). Eight parent–child dyads participated, with the remaining sample members participating alone. Tables 1 and 2 display the demographic characteristics and MTUAS outcomes, respectively.

**Table 1:** Demographic characteristics^a,b^

	Teen (*n* = 17)	Caregiver (*n* = 10)
Age, *M*(SD; range)	15.88 (.99; 14–17)	41.00 (7.96; 28–54)
**Gender, *n*(%)**		
Cisgender male	3 (17.6%)	1 (9%)
Cisgender female	14 (82.4%)	10 (91%)
**Race, *n*(%)**		
American Indian or Alaska Native	1 (5.95)	-
Black or African American	14 (82.4%)	7 (63.6%)
More Than One Race	1 (5.9%)	2 (18.2%)
White	-	2 (18.2%)
Prefer Not to Answer	1 (5.9%)	-
**Ethnicity, *n*(%)**		
Hispanic/Latinx	4 (23.5%)	1 (9.1%)
Non-Hispanic/Latinx	12 (706%)	9 (81.8%)
Prefer not to answer	1 (5.9%)	-
**Sexual orientation, *n*(%)**		
Heterosexual/Straight	13 (76.5%)	9 (81.8%)
Bisexual or Pansexual	4 (23.5%)	1 (9.1%)
**Highest level of education, *n*(%)**		
Some high school	17 (100%)	-
High school degree	-	1 (9.1%)
Some college	-	4 (36.4%)
College degree	-	2 (18.2%)
Business or technical school degree	-	1 (9.1%)
Attended graduate or professional school	-	1 (9.1%)
Graduate or professional school degree	-	1 (9.1%)
**Employment status, *n*(%)**		
Employed, full-time	-	7 (63.6%)
Employed, part-time	5 (29.4%)	2 (18.2%)
Not working, looking for work	-	1 (9.1%)
Student, part-time	-	2 (18.2%)
**Family income (US$), *n*(%)**		
Under $10,000	4 (23.5%)	1 (9.1%)
$10,000–39,999	-	4 (36.4%)
$40,000–$69,999	2 (11.8%)	2 (18.2%)
$70,000–99,999	-	1 (9.1%)
$100,000–129,000	2 (11.8%)	2 (18.2%)
Prefer not to answer/do not know	9 (53%)	-

^a^*M* mean, *SD* standard deviation

^b^Gender was assessed using 7 categories (cisgender male/female, transgender male/female, non-binary, not listed, prefer not to answer); the sample only identified as cisgender male/female

**Table 2:** Technology and smartphone usage and attitudes^a,b^

	Teen (*n* = 17)	Caregiver (*n* = 10)
Smartphone access	17 (100%)	NA
Use an app to manage a medical condition	2 (11.8%)	NA
Use an app to manage a mental health condition	1 (5.9%)	NA
**MTUAS usage**		
Smartphone usage	7.56 (1.38; several times a day)	7.76 (2.41; several times a day)
General social media usage	7.00 (1.99; several times a day)	5.34 (2.44; several times a week)
Internet searching	6.93 (2.11; once a day)	7.85 (2.89; several times a day)
Emailing	7.04 (1.43; several times a day)	7.58 (2.30; several times a day)
Media sharing	5.15 (2.12; several times a week)	5.50 (3.63; several times a week)
Text messaging	8.47 (1.29; once an hour)	8.10 (1.89; once an hour)
Video gaming	6.37 (3.09; once a day)	5.90 (2.69; several times a week)
Online friendships	2.47 (1.59; once a month)	3.13 (2.52; several times a month)
Social media friendships	4.79 (2.05; once a week)	4.69 (2.29; once a week)
Phone calling	6.50 (1.83; once a day)	7.25 (1.98; several times a day)
TV viewing	7.73 (2.00; several times a day)	6.30 (3.41; once a day)
**MTUAS attitudes**		
Positive attitudes toward technology	3.77 (.71; neither agree nor disagree)	3.90 (.62; neither agree nor disagree)
Anxiety about being without technology/dependence on technology	3.35 (1.02; neither agree nor disagree)	2.90 (1.01; disagree)
Negative attitudes toward technology	3.10 (.81; neither agree nor disagree)	2.93 (.91; disagree)
Preference for task switching	2.52 (.68; disagree)	2.75 (.72; disagree)

^a^*NA* not assessed, *MTUAS* media and technology usage and attitudes scale

^b^The Likert anchor is provided for the mean values of the MTUAS (e.g., a score of 7 on the usage scales ties to “several times a day”)

### Themes

Six themes emerged from the teens and caregivers. Five themes and their respective sub-themes are detailed below and displayed in Table 3; all data specific to pandemic-related experiences are reported elsewhere (Stiles-Shields et al., Under Review).

**Table 3:** Focus group themes, subthemes, and added data

**Themes**	**Subthemes**	**Examples**
Health and wellness concerns	Concerns of teens in our communities	All teens expressed mental health concerns in their communities from difficulties seen with depression (53%), stress (41%), anxiety (35%), and bullying/cyberbullying (29%) Caregivers expressed equal health-related concern around obesity/nutrition/lack of physical activity and mental health (each 70%)
	I’m concerned about my teen’s tech use	70% of caregivers reported concerns with their teens’ overuse of a smartphone and/or social media
	Adult check-ins happen, help	All teens reported parents check-in 47% of teens shared that their teachers, classrooms, or schools implement wellness efforts
	Open to seeking help	76.4% of teens expressed a willingness to seek help for reported concerns either from a professional or through an app, with 69% expressing caveats 40% of caregivers also expressed caveats around their teens seeking mental health treatment “[Parents] feel like it’s gonna be a reflection on them.” (caregiver of a 17-year-old)
Barriers	Barriers to in-person mental healthcare	20% of caregivers and 53% of teens identified shame and discomfort discussing mental health with people in their community as a barrier to healthcare “Mental health is something we don’t talk about in our community. It’s something that you just—you’re quiet about. Shame. Shame, pride, embarrassment…if the app could possibly help them break through the barriers that they have imposed on themselves in their mind that there’s embarrassment and shame in speaking to someone about it versus going in to speak to someone in person.” (caregiver of a 17-year-old)
	Barriers to smartphone use	71% of teens described how WiFi and service outages limited their use of smartphones, though many described this as a rare occurrence
	Hesitancy to use health and wellness apps	23.5% of teens were hesitant on whether they would use a mental health app consistently, while 40% on caregivers were hesitant about the possibility of their becoming dependent on an app used to improve their mood
Use of smartphones	Health and wellness	Teens used smartphones for health and wellness for activities such as exercise tracking (41%); meditation/stress control (47%); sleep monitoring (11.8%); accessing medical records (5.9%) and tracking time spent on the smartphone to improve digital wellbeing (5.9%)
	Logistics of use	Teen smartphone use was greatest at night (59%), followed by during the day (41%), in the morning (24%), or all the time (11.8%) “I’d say I probably use it most in the middle of the day because that’s when I get sidetracked from school.” (15-year-old)
Impact of smartphones	Increased attachment	76% of teens mentioned keeping their phones in close proximity, citing increased attachment to their smartphones “I’m not gonna lie to you. I never put mine down. I’m either havin’ my phone or my Apple watch at all times. If the battery runs down, I sit back and I grab my Samsung phone…I always have it. I wasn’t used to havin’ a smartphone. Now that I have one, I think that might be goin’ a little bit overboard.” (14-year-old)
	Mixed effects on mood	Some teens felt smartphones made them feel “sad” or “lonely.” This feeling was often attributed to heavy social media use
	Mixed effects on health	82.4% of teens described smartphones impacting their health, with 29.4% citing negative and 52.9% citing positive effects. Negative health effects of smartphones shared included loss of sleep, stress, and fatigue. Positive health effects from smartphone use stemmed from applications that benefited teens’ wellbeing. These apps focused on fitness, meditation, and even water intake
	Causing distraction	70.6% of teens spoke heavily about their phones keeping them distracted from schoolwork, family time, or sleep
	Desire for changes in use	41.2% of teens and 10% of caregivers mentioned teens wanting to change how much they used their phones
Opinions and suggestions for mHealth apps	Pros	80% of caregivers endorsed a preference for their teens to use an app to manage behavioral health over traditional, face-to-face treatment. Similarly, all teens stated that mental health-focused apps would be beneficial
	Cons	80% of parents shared worries such as the possibility of their teens not fully understanding the tool, apprehension about their teen sharing personal information, and fear of their teen becoming dependent on an mHealth app “I can see somethin’ maybe helpin’ her, but I wouldn’t want her to be dependent on it. It would be good if there was something that would give her strategies of betterin’ her mood when she’s feelin’ down, but I wouldn’t want her to be dependent on an app.” (Caregiver of a 17-year-old)

#### Health and Wellness Concerns

##### Concerns of Teens in Our Communities

All teens expressed mental health concerns and described other situations in their communities, including depression (53%), stress (41%), anxiety (35%), and bullying/cyberbullying (29%). A 16-year-old teen stated: “You start going through hard things, and you start noticing the world and that life isn’t easy… It’s up to you if you can handle them and fix them and solve them, or if it’s too much… I think that’s the time, once you get depressed, around teenage age.” Thirty-five percent of teens associated mental health concerns with remote learning and 23% with social media use. Other concerns included eating habits and lack of fitness (29%), anger, loneliness, early pregnancy, lack of self-care, peer pressure, and substance use (35%).

Caregivers expressed equal health-related concern around obesity/nutrition/lack of physical activity and mental health (each 70%). A caregiver of a 17-year-old noted: “Where I live there’s no real place to eat quality food. There’s no place that sells healthy food, and it’s hard to preach eating healthy at home when there’s a place just down the street that sells burgers and fries.” All caregivers associated mental health concerns to the effects of COVID-related isolation. Connecting these points, a caregiver of a 17-year-old stated: “I think obesity and mental health” as top health issues affecting teens in their community, “because our children are locked in the house.” Caregivers (20%) also expressed concerns about misinformation on social media and lack of communication between parents and children.

Safety was a consistent community concern, mentioned by 23.5% of teens and 70% of caregivers. A 16-year-old described “seeing somebody be a victim of violence, especially if you know that person, like some of my friends. That just really puts them in a bad place. They just become closed off, and just go into a depressed state.” Caregivers expressed concern over community and police violence, as well as a changing feeling of community: “It feels like we don’t have that sense of community anymore, which is sad. A community will ease the pain of everything. If your neighbor needs help, it’s always nice when there’s somebody there to help ‘em out. Now, everything is kinda so individualized” (Caregiver of a 16-year-old). All caregivers described their teens as having many emotional needs, often stemming from the uncertainty and instability brought on by current events like COVID and violence against Black communities.

##### I’m Concerned About My Teen’s Tech Use

Caregivers (70%) reported concerns with their teens’ overuse of a smartphone and/or social media and its implications, namely distraction from other activities. A caregiver of a 15-year-old stated: “it may affect his overall feeling or, you know, wellbeing just being so glued to that device and not being social and active.” A caregiver of a 16-year-old expressed similar sentiments about social media: “[My daughter] says there’s nothing else to do, so if she ain’t on [her smartphone] for school, she’s on there for social—every day, all day.” Half of caregivers reported concerns with technology granting their teens too much access to misinformation or dangerous, fear-stoking content. A caregiver of a 17-year-old implicated social media in particular: “That’s the other sad part about social media and smartphones, is that even the news it’s just instilling fear in people, period. The older ones, the baby boomers, and even the young people.”

##### Adult Check-ins Happen, Help

All teens and caregivers expressed support for trusted-adult check-ins about mental health. About half of the teens (53%) shared that at least one parent checked-in with them. While one 16-year-old expressed ambiguity about parental check-ins: “My family’s important to me, but… I just don’t really open up,” most teens shared that they feel comfortable with parents asking how they are feeling and that they have an open communication with them. Two teens expressed that they feel more comfortable talking to their parents or siblings over their doctor. Most caregivers (90%) checked-in with their teen, whether daily or when they noticed a change in behavior (e.g., “It’s not a everyday thing, but sometimes I can look at her and tell somethin’ is botherin’ her” [Caregiver of a 16-year-old]).

About half of teens (47%) shared that their teachers, classrooms, or schools implemented wellness efforts. These included mental health surveys and referrals to a school support group or meeting with a counselor, using apps, class bonding activities, or teachers simply checking in. Teens expressed appreciation for these check-ins. A 17-year-old reported: “[Counselors will] ask you in a survey or during school or virtual. …They'll say, ‘Do you wanna be joined into this group where we talk about our problems when we do meditation’; I feel like that's really helpful, especially for everyone that's going through something at home and going to school…”.

##### Open to Seeking Help

Most teens (76.4%) expressed willingness to seek help for reported concerns either from a professional or through an app, while three-quarters (69%) noted caveats: (1) The referral must come from a trusted source, (2) the help must be directly offered to them versus them having to search for it, (3) follow-through depends on the teen’s personality and openness to talk and how much mental health symptoms are affecting them, (4) teens may be more willing to seek help for certain concerns (e.g., anxiety over depression), (5) teens may be more willing to seek help through app versus in-person due to privacy or wanting to be independent, and (6) teens who seek help may not be able to get it.

Caregivers reported support for counseling and therapy services and education on the long-term effects of not taking care of one’s body (nutrition, exercise, etc.). Two caregivers described therapy as helpful (individually or with their teen). Nearly half of caregivers (40%) expressed caveats, including the following: (1) Potential stigma around their teens seeking mental health treatment (“[parents] feel like it’s gonna be a reflection on them”), (2) the provider must be someone the teen feels they can trust, (3) there must be transparency in the type of information being collected and what it is being used for, and (4) If it is a group therapy treatment, it is important that the others be “like-minded kids.”

#### Barriers

##### Barriers to In-person Mental Healthcare

Three-quarters of teens and 40% of caregivers described significant barriers to receiving in-person mental healthcare. Half of these teens (53%) and 20% of those caregivers described shame and discomfort discussing mental health within their community as a barrier to healthcare. A caregiver of a 17-year-old stated: “Mental health is something we don’t talk about in our community. It’s something that you just—you’re quiet about. Shame. Shame, pride, embarrassment.” Other barriers to receiving in-person mental health care included distrust in healthcare providers and medical institutions and minimization of mental health problems by family members.

##### Barriers to Smartphone Use

About three-quarters of teens (71%) described Wi-Fi and service outages that limited their use of smartphones, though some described this as a rare occurrence. Most teens described brief interruptions in service and Wi-Fi based on location and weather; however, one 17-year-old described financial difficulties leading to loss of Wi-Fi, stating, “Some of the times, my parents are not able to pay off the Wi-Fi, so I can't access the internet. I basically just have to start using the messages or just calling someone.” Only 30% of caregivers described Wi-Fi and service outages as barriers to smartphone use. A caregiver of a 15-year-old stated that her teen does not have a service plan: “She doesn't have a data plan, or a phone plan, so she uses a Wi-Fi function at home. Or whenever she's somewhere that can use Wi-Fi. When she is able to hold a job that she can pay for herself, then she can get a plan.” Additionally, 42% of teens and most caregivers (70%) described household restrictions on smartphone use. Rules included banning phone use during school hours, at night, and at the dinner table.

##### Hesitancy to Use Health and Wellness Apps

Most teens (59%) and caregivers (80%) described hesitancy to use smartphones for health and wellness. Teens described hesitance because they felt that they were not likely to use such an app consistently (23.5%), their smartphone was a cause of poor mental health outcomes (11.8%), they believe that human interaction is important for mental health (11.8%), they have no health problems (11.8%), and/or they do not believe such an app would accurately reflect their state of health (11.8%). One 17-year-old described her disbelief that a health or wellness app would be a long-term solution, stating, “Some people are probably just download it and start using it heavy the first couple weeks. Then some would probably forget about it.”

Some caregivers (40%) worried about their children becoming dependent on an app to improve their mood (e.g., “What if you lose your phone and you don’t have this app? Now you’re sad because you don’t have your phone, and you don’t have an app. Now you [are] just on a destructive path because you were so dependent on this app to tell you that your mood was bad and to give you strategies to make it better” [Caregiver of a 17-year-old]). Caregivers also described distrust in technology, lack of teen buy-in, and worries that the app would keep teens from confiding in their caregivers as reasons for their hesitancy.

#### Use of Smartphones

Responses on the MTUAS (Table 2) indicated that on average, both teens and caregivers used their smartphones several times a day. Teens also reported that they text messaged several times an hour, while social media use and emailing occurred several times a day, on average.

##### Health and Wellness

Beyond use for functions of daily living, communication, and entertainment, teens used smartphones for health and wellness for exercise tracking (41%), meditation/stress control (47% teens), sleep monitoring (11.8% teens), accessing medical records (5.9%), and tracking time spent on the smartphone to improve digital well-being (5.9%).

##### Logistics of Use

Teen smartphone use was greatest at night (59%), followed by during the day (41%), in the morning (24%), or all the time (11.8%). Several teens described boredom during class time as a reason for daytime use, with a 15-year-old stating, “I’d say I probably use it most in the middle of the day because that’s when I get sidetracked from school.”

#### Impact of Smartphones

##### Increased Attachment

Most teens (76%) mentioned keeping their phones in close proximity. A 17-year-old stated, “I keep my phone right next to me at all times,” and a 14-year-old further emphasized use: “I’m not gonna lie to you. I never put mine down.” Caregivers also noted high use and proximity (e.g., “Yes, I agree. My daughter—she’s on it 24/7. She says there’s nothing else to do, so if she ain’t on it for school, she’s on there for social—every day, all day” [Caregiver of 16-year-old]). Phone proximity led to discussions of personification of smartphones. Indeed, a 14-year-old stated: “I think my smartphone is, like, a part of me. So I feel more comfortable sharing with my smartphone than I’d share with a person in real life.” A 14-year-old directly attributed her attachment as a reason she has found an mHealth app helpful for her mood: “the way I’m imaging it right now is as if that app was a person—a friend. And although I feel like I can talk to my friends, I don’t think I can share everything. So, if there’s an app that helps me while still keeping it personal where I can write whatever I want, or do whatever I want without fearing, like, judgment or anything, then it's great.”

##### Mixed Effects on Mood

All teens stated that their smartphones affected their mood. Some teens felt smartphones made them feel “sad” or “lonely.” This feeling was often attributed to heavy social media use. A 17-year-old stated: “It's degrading because social media, they are so quick to judge people. That's why you have to be careful with that.” Positive effects of smartphone use were also noted. Teens spoke of the ways apps including YouTube, TikTok, Instagram, and TV or music streaming applications brightened their moods from the various content they provided. A 16-year-old stated:

I use my smartphone for YouTube, or Netflix, HBO, and so on or Instagram and Snapchat and more apps that we usually use that are very common. I usually use those when it comes to when I'm down. Sometimes I use them, too, to calm myself down because sometimes I just overstress, or I'm just feeling upset over a particular thing, or something that's bothering me too much. I use it to just get my mind off the situation that I'm goin' through.

Teens implied that social media provides temporary solutions, while apps and digital tools directly target coping skills. A 16-year-old explained:

I use YouTube most of the time because I feel like it just helps instead of apps. I think YouTube gives me ideas of what I should do. How to calm myself down. Sometimes, you can even put a video where they tell you like, "Okay. You count down from this number to this number, or you breathe in and you breathe out." I feel that helps for me a lot, when I'm stressing or feeling anxious for some particular things.

Diaphragmatic breathing and mindfulness skills were frequently endorsed by teens. A 17-year-old stated: “I used to have an app… It was really for meditation and [to] make sure I do my breathing exercises… It was really helpful when I used to overthink or get too down.”

##### Mixed Effects on Health

Most teens (82.4%) described smartphones impacting their health, with 29.4% citing negative and 52.9% citing positive effects. The negative health effects of smartphones included loss of sleep, stress, and fatigue. Within these discussions, several teens spoke of heavy phone use at night, leading to a loss of sleep. A 14-year-old explained: “I would say at night– very late at night – from 11 p.m. to 3 a.m. I know it’s not good, but it’s hard for me to go to sleep, so I get distracted on my phone.” 

Positive health effects from smartphone use stemmed from applications that benefited teens’ well-being. These apps focused on fitness, meditation, and even water intake. Four teens specifically described experiences using apps that helped with relieving stress. About a quarter of teens (23.6%) brought up the app, Headspace, and how its features for meditation and sleep were helpful (e.g., “It has meditation things and helps me sleep better, even though I don’t really sleep too much, but I think without it, it would have been much more harder to fall asleep. It just reduces stress sometimes when I feel overwhelmed” [Teen, 14]). Teens also introduced the idea of apps being helpful when introduced by a trusted source (e.g., teachers).

##### Causing Distraction

Most teens (70.6%) described smartphones as a distraction from schoolwork, family time, or sleep. A 15-year-old agreed with another participant about phone use during family time being considered rude: “Yeah, same thing with the family time. I don't be on my phone. And then toward like night time, 'cause I be up all night. My mom, she will tell me sometimes to get off my phone when it's really, really late. Mainly just when it's family time.” However, multiple teens went on to describe using smartphones to distract themselves from emotions. A 14-year-old explained: “When I’m having a problem, I try to use my phone to not think about the problem anymore. Trying to calm down sometimes when I’m a little bit anxious or stressed, I’m worried about something.”

##### Desire for Changes in Use

About half of teens (41.2%) and 10% of caregivers mentioned teens wanting to change how much they used their phones. A 17-year-old explained: “Sometimes, I feel like it's not good for my mental health or the mood I'm in. Sometimes, I just wanna be outside or just away from my phone and just take a break from it.” Social media was repeatedly noted as a reason for wanting to change smartphone usage. A 15-year-old explained how she already changed her phone use by removing social media:

This summer, my sister and I signed a personal contract between the two of us saying that we wouldn’t use social media. I don’t have any of that on my phone right now. My phone is purely to call people and take pictures now… I just feel I have so many artistic visions and so many things that I want to do. I want to learn how to play my guitar better or I have things that I want to make. I’m not really an artistic person, but I feel like—the only reason I don’t—is ‘cause I’m on social media. I don’t like it because I don’t enjoy my time on social media as much as I enjoy my time doing other things. It’s an unnecessary distraction.

A caregiver echoed this experience, describing her 15-year-old as “self-policing” when it came to her smartphone.

#### Opinions and Suggestions for mHealth

##### Pro

Most caregivers (80%) endorsed a preference for their teens to use an app to manage behavioral health over traditional, face-to-face treatment. Caregivers believed that their teens’ smartphone use and habits made them more likely to use apps as a resource (e.g., “I think they'll be more inclined to use an app, because that's just what they do. That's how they communicate today.” [Caregiver of 16-year-old]). Caregivers also stated that the use of apps may overcome potential stigma to seeking care: “I just think the smart app is easier… because some people are embarrassed. They think mental health or anything like that is an embarrassment, which it really isn’t. I think being able to get their feet wet on the app would probably be better. Then they may switch over to the extra help” (Caregiver of a 17-year-old).

All teens stated that such mental health-focused apps would be beneficial (e.g., “it would be a way to keep myself on track so that I can, you know, analyze how I’m feeling and be able to express that in a healthy way” [Teen, 14]). They also emphasized the comfort they already have with smartphones and the lack of perceived judgment: “If there’s an app that helps me while still keeping it personal where I can write whatever I want, or do whatever I want without fearing, like, judgment or anything, then it's great” (Teen, 14). While teen participants expressed support for mHealth tools focusing on mood management, they also noted that it can be hard for such tools to reach teens:

I think that teens would use it. It’s just that you have to get the knowledge out there to the teens. A lotta times we don’t use apps because we don’t really know… I’m just only sayin’ for the students that go to my high school, I think that it would be a benefit because it’s a lotta teens that’s really hurtin’ right now. You know how sometime you really don’t know who to turn to. (Teen, 14)

Nearly all caregivers (90%) supported the use of an mHealth app focused on mood management. Indeed, multiple parents voiced that a mood app might provide a new way to approach and engage their teens about their mood. For example:

My daughter—she have a lot of mood changes. She’s a girl [laughter]. Sometimes, I think something like that would be good for me because it’ll give me a way to come at her, to talk to her, to get her to open up about her feelings, ’cause sometimes, me just asking or whatever, she blowin’ me off like, ain’t nothin’ wrong, it’s okay… I’m thinkin’ maybe if it was a app that give different directions to go at, it would be more ways… for my teen to open up to me about her feelings… I gotta push and shove just to get it out of her, so I’m thinkin’ different ways and better ways to approach would be a good way to—for her to open up to me more. (Caregiver of a 16-year-old)

Caregivers also viewed apps as a means to increase skill building for mood management: “I would be all for mental health training. … I wish when I was younger I had that. If that was something that happened now for my daughter, I would be thankful for that” (Caregiver of a 17-year-old).

All caregivers and teens stated that they supported the use of mHealth for broader wellness and as a source for credible health information. Teens described a desire to use apps as a source of motivation for improved wellness: “I think I would use it because it could probably give me good advice and also help me throughout life and can motivate me to do better.” (Teen, 16). Teens also voiced a desire for guidance or structure to improve wellness. For example: “I think it would be useful for me ‘cause it seems that as you get older, you’re expected to…take care of yourself. You’re s’posed to know how many hours to go to bed, what’s the right foods to eat, how many times to exercise in a day. Having a app to help monitor that with me, yeah. I think that would be a help in my life” (Teen, age 16). Caregivers also described encouraging their teens to use apps for wellness. Some went on to say that having access to their teens’ wellness data helps them with structuring their care of their children: “It’s actually useful because sometimes my youngest daughter, she wakes up for school and she’s cranky and she’s tired… I can tell if she bypassed the sleep timer and continued to use her phone… It actually does help” (Caregiver of a 17-year-old).

##### Con

Despite caregivers expressing their beliefs that their teens would prefer the use of a smartphone app to manage their behavioral health over traditional treatment, they also expressed concerns about their teens’ use and possible reliance on apps used to benefit mental health. Caregivers (80%) shared varying worries, including the following: the possibility of their teens not fully understanding the tool, apprehension about their teen sharing personal information, and fear of their teen becoming dependent on an mHealth app. When queried about an app that provided their teens with tips and skills for improving their mental health, a caregiver of a 16-year-old stated: “[If] she has something that’s a problem, talk to me and we’ll try to, both of us, to resolve it.” The hesitancy that caregivers expressed about their teens confiding in an app instead of their loved ones led to previously described discussions of concerns about their teens’ potential dependence on an app that worked to improve their mood (e.g., “No. I don’t think I would be okay with that type of app. I wouldn’t want her to be depending on pullin’ up the app to change her mood. That’s somethin’ that she would need to do internally” [Caregiver of a 17-year-old]).

All teens voiced apprehension or possible concerns about starting with and continuously using mHealth apps. A common concern was perpetuation of loneliness. A 17-year-old spoke about their peers coping with depression and anxiety by writing in their notebooks, instead of seeking treatment; when asked if an app might provide the same benefits, they explained: “I feel like it could be a possibility—I mean, it depends. If the smartphone is what is giving this person anxiety and depression because of the people on social media, they could not wanna be on their phone.” Other teens stated that support from family or friends might be preferred to using an app (e.g., “Because, a app, I don’t feel like it knows you enough to know what make you happy or make you sad or somethin’ like that. I think it’s better for a person to help with your mood than [an] app” [Teen, 17]).

## Discussion

To improve the design and engagement beyond the pandemic, this study used HCD methodologies to identify the mHealth preferences and needs of teens with SCN (Bounds et al., 2020). Teens and caregivers reported frequent smartphone and broader technology use on the MTUAS. This self-reported use was corroborated by their qualitative reports, which centered upon their health and wellness concerns for teens in their communities, barriers to care, and the use, ubiquity, and impacts of smartphones.

Teens and their caregivers are acutely aware of mental and behavioral health burdens disproportionately placed upon teens in their communities, from community violence to obesity to depression. As a generation who grew up with digital devices (Psihogios et al., 2022), many expressed an openness to mhealth due to the ubiquity, comfort with, and access to smartphones. Yet, the familiarity and frequency of smartphone use also presents potential barriers. Indeed, while smartphones may be an avenue to access evidence-based screening and intervention tools, they are also seen as a distraction, overused, and as a potential delivery mechanism for problematic online experiences with misinformation or on social media. mHealth designed for teens with SCN therefore must not only address frequently cited barriers, such as data and privacy concerns—including privacy from caregivers (Cavazos-Rehg et al., 2020; Stiles-Shields et al., 2017), but also address the complexity of using a smartphone to benefit mental health. This complexity lies in the need to moderate exposure to potential harm caused by simultaneously using other smartphone tools.

Mental healthcare-related stigma is a well-known phenomenon, particularly in communities facing hardships perpetuated through systemic racism (Misra et al., 2021). Despite the opportunity for privacy potentially afforded by digital delivery, stigma may be reflected as an mHealth barrier via caregiver concern for dependency. Caregivers expressed concern that mental health-targeted mobile tools could foster a need for ongoing use to regulate their teens’ moods, facilitating a dependency. Such concerns were absent from the enthusiastic response to mHealth targeting behavioral health concerns (e.g., sleep). Potential mHealth designers must expand on established co-design methods with teens (Fleming et al., 2016; Hetrick et al., 2018; Thabrew et al., 2018) to also collaborate with caregivers to provide approachable, engaging, and concise psychoeducation about the role of mental health-focused mHealth (e.g., skills-based, time-limited use). However, insights from participants also highlighted an existing, leverageable resource to encourage exposure and potential comfort with mental health-targeted apps: trusted adults in the community. Consistent with a growing literature for the benefits of concurrent human support in the use of digital tools (Lattie et al., 2022), all participants expressed an openness to adults checking-in about teen mental health and making recommendations for resources (e.g., teachers suggesting an app). Approaching and engaging teens with SCN—and their caregivers—is likely supported through community-engaged approaches that include representative stakeholders as collaborators from design inception through open deployment with community teens (Noel, 2020; Stiles-Shields et al., 2022).

The current findings should be considered in light of specific limitations. Eligibility criteria required participants to have self-reported smartphone use within the week prior to enrollment. Additionally, while most themes can be discovered within three to six focus group sessions (Guest et al., 2017), the small sample size should be considered with caution given the potential to be less representative of the general population of teens with SCN, particularly in view of potential variability in smartphone use. As such, it is unclear how these findings extend to teens with SCN with less frequent or inconsistent smartphone access. Relatedly, self-reported smartphone and broader technology use on the MTUAS may not accurately reflect the use and frequency of daily teen behaviors (Ellis et al., 2019). The participants were also recruited remotely within the context of the pandemic; it is unclear how their reports generalize considering the use of virtual recruitment (e.g., “zoom fatigue” during remote learning). Future research should continue to specifically engage youth with SCN and their caregivers to ensure representative input in mHealth and broader digital health development and dissemination. Furthermore, to promote inclusive representation, future research should consider multiple approaches to collaboration. Indeed, some teens and caregivers may prefer remote sessions (e.g., Zoom), whereas others might not have this preference and/or access to the necessary devices and consistent broadband internet.

This current study identified teen and caregiver-reported perspectives about mHealth in the context of emerging from the COVID-19 pandemic. Nuanced barriers to mHealth, including ongoing negative perspectives about current smartphone use, may impact the engagement of teens with SCN if left unaddressed. Continued and deliberate collaboration with teens and their caregivers across the design and deployment process will be critical to ensuring mHealth may reach and serve teens with SCN.

## References

[CR1] Adkins EC, Zalta AK, Boley RA, Glover A, Karnik NS, Schueller SM (2017). Exploring the potential of technology-based mental health services for homeless youth: A qualitative study. Psychological Services.

[CR2] Anderson, M., & Jiang, J. (2018). *Teens, social media & technology 2018*. Pew Research Center. Retrieved November 18, 2021, from https://www.pewinternet.org/2018/05/31/teens-social-media-technology-2018/

[CR3] Anderson-Lewis C, Darville G, Mercado RE, Howell S, Di Maggio S (2018). mHealth technology use and implications in historically underserved and minority populations in the United States: Systematic literature review. JMIR mHealth and uHealth.

[CR4] Baum F, MacDougall C, Smith D (2006). Participatory action research. Journal of Epidemiology and Community Health.

[CR5] Benuto LT, Singer J, Newlands RT, Cases JB (2019). Training culturally competent psychologists: Where are we and where do we need to go?. Training and Education in Professional Psychology.

[CR6] Bounds DT, Winiarski DA, Otwell CH, Tobin V, Glover AC, Melendez A, Karnik NS (2020). Considerations for working with youth with socially complex needs. Journal of Child and Adolescent Psychiatric Nursing: Official Publication of the Association of Child and Adolescent Psychiatric Nurses, Inc.

[CR7] Braun V, Clarke V (2006). Using thematic analysis in psychology. Qualitative Research in Psychology.

[CR8] Cavazos-Rehg P, Min C, Fitzsimmons-Craft EE, Savoy B, Kaiser N, Riordan R, Krauss M, Costello S, Wilfley D (2020). Parental consent: A potential barrier for underage teens’ participation in an mHealth mental health intervention. Internet Interventions.

[CR9] Ellis DA, Davidson BI, Shaw H, Geyer K (2019). Do smartphone usage scales predict behavior?. International Journal of Human-Computer Studies.

[CR10] Figueroa CA, Aguilera A (2020). The Need for a mental health technology revolution in the COVID-19 pandemic. Frontiers in Psychiatry / Frontiers Research Foundation.

[CR11] Fleming TM, Bavin L, Stasiak K, Hermansson-Webb E, Merry SN, Cheek C, Lucassen M, Lau HM, Pollmuller B, Hetrick S (2016). Serious Games and gamification for mental health: Current status and promising directions. Frontiers in Psychiatry / Frontiers Research Foundation.

[CR12] Fortney JC, Pyne JM, Turner EE, Farris KM, Normoyle TM, Avery MD, Hilty DM, Unützer J (2015). Telepsychiatry integration of mental health services into rural primary care settings. International Review of Psychiatry.

[CR13] Guest G, Namey E, McKenna K (2017). How many focus groups are enough? Building an evidence base for nonprobability sample sizes. Field Methods.

[CR14] Harris PA, Taylor R, Thielke R, Payne J, Gonzalez N, Conde JG (2009). Research electronic data capture (REDCap): A metadata-driven methodology and workflow process for providing translational research informatics support. Journal of Biomedical Informatics.

[CR15] Hetrick SE, Robinson J, Burge E, Blandon R, Mobilio B, Rice SM, Simmons MB, Alvarez-Jimenez M, Goodrich S, Davey CG (2018). Youth codesign of a mobile phone app to facilitate self-monitoring and management of mood symptoms in young people with major depression, suicidal ideation, and self-harm. In JMIR Mental Health.

[CR16] Hill, C., Molitor, M., & Ortiz, C. (2015). *Racism and inequity are products of design. They can be redesigned*.

[CR17] Hilty DM, Crawford A, Teshima J, Nasatir-Hilty SE, Luo J, Chisler LSM, Gutierrez Hilty YSM, Servis ME, Godbout R, Lim RF, Lu FG (2021). Mobile health and cultural competencies as a foundation for telehealth care: Scoping review. Journal of Technology in Behavioral Science.

[CR18] Hilty DM, Zalpuri I, Torous J, Nelson E-L (2021). Child and adolescent asynchronous technology competencies for clinical care and training: Scoping review. Families, Systems & Health: THe Journal of Collaborative Family Healthcare.

[CR19] IDEO. (2015). *The Field Guide to Human-centered Design: Design Kit*. IDEO.

[CR20] International Organization for Standardization. (2019). *ISO 9241--210: 2019 (en) Ergonomics of human-system interaction—Part 210: Human-centred design for interactive systems*.

[CR21] Iskander, N. (2018). Design thinking is fundamentally conservative and preserves the status quo. *Harvard Business Review*, *5*.

[CR22] Kendi, I. X. (2019). *How to be an antiracist*. Random House Publishing Group.

[CR23] Kumar D, Hemmige V, Kallen MA, Giordano TP, Arya M (2019). Mobile phones may not bridge the digital divide: A look at mobile phone literacy in an underserved patient population. Cureus.

[CR24] Lange-Maia BS, De Maio F, Avery EF, Lynch EB, Laflamme EM, Ansell DA, Shah RC (2018). Association of community-level inequities and premature mortality: Chicago, 2011–2015. Journal of Epidemiology and Community Health.

[CR25] Lattie EG, Stiles-Shields C, Graham AK (2022). An overview of and recommendations for more accessible digital mental health services. Nature Reviews Psychology.

[CR26] Misra S, Jackson VW, Chong J, Choe K, Tay C, Wong J, Yang LH (2021). Systematic review of cultural aspects of stigma and mental illness among racial and ethnic minority groups in the United States: Implications for interventions. American Journal of Community Psychology.

[CR27] Neary M, Schueller SM (2018). State of the field of mental health apps. Cognitive and Behavioral Practice.

[CR28] Noel, L. -A. (2020, July 3). My manifesto towards changing the conversation around race, equity and bias in design. *Medium*. https://medium.com/future-of-design-in-higher-education/9-steps-towards-changing-the-conversation-around-race-equity-and-bias-in-design-304242194116

[CR29] Noorbergen TJ, Adam MTP, Roxburgh M, Teubner T (2021). Co-design in mHealth Systems development: Insights from a systematic literature review. AIS Transactions on Human-Computer Interaction.

[CR30] Patton, M. Q. (1990). *Qualitative evaluation and research methods, 2nd ed*. *2*, 532.

[CR31] Pierce JB, Harrington K, McCabe ME, Petito LC, Kershaw KN, Pool LR, Allen NB, Khan SS (2021). Racial/ethnic minority and neighborhood disadvantage leads to disproportionate mortality burden and years of potential life lost due to COVID-19 in Chicago, Illinois. Health & Place.

[CR32] Psihogios AM, Lane-Fall MB, Graham AK (2022). Adolescents are still waiting on a digital health revolution: Accelerating research-to-practice translation through design for Implementation. JAMA Pediatrics.

[CR33] Psihogios AM, Stiles-Shields C, Neary M (2020). The needle in the haystack: Identifying credible mobile health apps for pediatric populations during a pandemic and beyond. Journal of Pediatric Psychology.

[CR34] Rosen LD, Whaling K, Carrier LM, Cheever NA, Rokkum J (2013). The media and technology usage and attitudes scale: An empirical investigation. Computers in Human Behavior.

[CR35] Safavi K, Mathews SC, Bates DW, Dorsey ER, Cohen AB (2019). Top-funded digital health companies and their impact on high-burden, high-cost conditions. Health Affairs.

[CR36] Schueller SM, Hunter JF, Figueroa C, Aguilera A (2019). Use of digital mental health for marginalized and underserved populations. Current Treatment Options in Psychiatry.

[CR37] Stanford d.School. (2010). *An introduction to design thinking PROCESS GUIDE*. Institute of Design at Stanford.

[CR38] Stiles-Shields C, Cummings C, Montague E, Plevinsky JM, Psihogios AM, Williams KDA (2022). A call to action: Using and extending human-centered design methodologies to improve mental and behavioral health equity. Frontiers in Digital Health.

[CR39] Stiles-Shields C, Montague E, Lattie E, Kwasny MJ, Mohr DC (2017). What might get in the way: Barriers to the use of apps for depression. Digital Health.

[CR40] Stiles-Shields C, Potthoff LM, Bounds DT, Burns MTS, Draxler JM, Otwell CH, Wolodiger ED, Westrick J, Karnik NS (2020). Harnessing phones to target pediatric populations with socially complex needs: Systematic review. JMIR Pediatrics and Parenting.

[CR41] Stiles-Shields, C., Reyes, K. M., Lennan, N., Zhang, J., Archer, A., Julion, W. A., & Shalowitz, M. U. (Under Review). Community teens’ COVID-19 experience: Implications for engagement moving forward. *Clinical Practice in Pediatric Psychology.* 10.1007/s10880-023-09975-z PMC11174976 37803094

[CR42] Substance Abuse and Mental Health Services Administration (SAMHSA). (2014). *Results from the 2013 National survey on drug use and health: Summary of findings*. http://www.samhsa.gov/data/sites/default/files/NSDUHresultsPDFWHTML2013/Web/NSDUHresults2013.pdf 27656739

[CR43] Thabrew H, Fleming T, Hetrick S, Merry S (2018). Co-design of ehealth interventions with children and young people. Frontiers in Psychiatry / Frontiers Research Foundation.

[CR44] Winters N, Langer L, Nduku P, Robson J, O’Donovan J, Maulik P, Paton C, Geniets A, Peiris D, Nagraj S (2019). Using mobile technologies to support the training of community health workers in low-income and middle-income countries: Mapping the evidence. BMJ Global Health.

